# Synthesis of Gold Nanoparticle Stabilized on Silicon Nanocrystal Containing Polymer Microspheres as Effective Surface-Enhanced Raman Scattering (SERS) Substrates

**DOI:** 10.3390/nano10081501

**Published:** 2020-07-31

**Authors:** Guixian Zhu, Lin Cheng, Gannan Liu, Lianqing Zhu

**Affiliations:** 1 School of Instrument Science and Opto-Electronics Engineering, Beijing Information Science and Technology University, Beijing 100192, China; chenglin51720@163.com (L.C.); 17810257075@163.com (G.L.); 2 School of Instrument and Opto-Electronics Engineering, Hefei University of Technology, Hefei 230009, China

**Keywords:** SERS, gold nanoparticle, silicon nanocrystal containing polymer microspheres, 4-mercaptopyridine, pH value

## Abstract

Developing ideal surface-enhanced Raman scattering (SERS) substrates is significant in biological detection. Compared with free non-aggregated noble metal nanoparticles, loading metal nanoparticles on a large matrix can achieve a higher SERS effect due to the existence of many “hot spots”. A novel SERS substrate with intense “hot spots” was prepared through reducing gold ions with silicon nanocrystal containing polymer microspheres. The substrate exhibits high SERS sensitivity with an enhancement factor of 5.4 × 10^7^. By applying 4-mercaptopyridine as a Raman reporter, the developed SERS substrate can realize measurement of pH values. The intensity ratio of 1574 to 1607 cm^−1^ of 4-mercaptopyridine showed excellent pH sensitivity, which increased as the surrounding pH increased. With good stability and reliability, the pH sensor is promising in the design of biological detection devices.

## 1. Introduction

Surface-enhanced Raman scattering has been continuously investigated in chemical and biological detection [1,2,3,4]. The localized surface plasmon resonance of noble metal nanoparticles generates strong electromagnetic field enhancements, which determines the surface-enhanced Raman scattering (SERS) effect [5,6]. The enhancement factor (EF) plays a significant role in SERS applications, which could be enhanced by developing a structure with aggregated noble metal (such as gold and silver) nanoparticles. It is shown that nanoparticles with a sub-10 nm gap distance can achieve a 10^10^ SERS enhancement effect on molecules [7].

The loading of metal nanoparticles within large scale matrices has attracted wide interest because of the improvement of matrices’ mechanical strength and reduction of nanoparticle aggregation [8,9,10]. Various materials have been used as supported matrices, such as silica spheres [11], polymers [12,13], polystyrene microspheres (PS) [6], and so on. For large porous silica, parts of noble metal nanoparticles are embedded in the core part of silica, which cannot capture target molecules effectively compared with those on the surfaces. For solid PS, chemical modification of nanoparticles shows low modification efficiency and stability compared with in situ growth of nanoparticles, which are embedded in the matrix. The preparation of highly sensitive SERS substrates remains a challenge. Among these materials, silicon-based materials have attracted interest because of low-loss optical resonances in the visible region, well-controlled structure, and reducibility to noble metal ions. Mostly 2D SERS-active substrates are investigated for previous reports [14,15,16]. For example, Syed Hamad et al. have reported gold/silver-embedded periodic surface structures on crystalline silicon for SERS platforms [15]. Compared with 2D substrates, 3D silicon-based SERS substrates can expand the scope of application areas [13,17]. In order to obtain high scale silicon-derived particles, we reported a one-step approach to synthesize fluorescent silicon nanocrystal containing polymer microspheres (SiPS) by controlling the reaction between aminosilane and L-ascorbic acid [18]. The reaction can also be applied on the surface of a solid template to form silicon nanocrystal embedded polymer film. Due to the existence of L-ascorbic acid, the microcapsules are able to reduce noble metal ions to generate metal nanoparticles inside.

pH sensing is significant in chemical and biomedical detection. A SERS-based pH sensor is composed of a SERS substrate and a pH sensitive molecule. Some weak acids have been applied as a pH-probe, such as 4-mercaptobenzoic acid (4-MBA) [19], 4-mercaptopyridine (4-MPy) [20], and aminothiophenol (ATP) [21]. Among them, 4-MPy is the most frequently applied molecule, due to its sensitive pH response, high Raman signal, and strong affinity with the metal nanoparticles.

In this work, the new SERS substrate, composed of gold nanoparticle stabilized silicon nanocrystal containing polymer microspheres, was successfully prepared. Silicon nanocrystal embedded hollow microspheres were synthesized successfully by applying PS as solid templates. In order to obtain SERS-active materials, silicon nanocrystal embedded hollow microspheres were mixed with noble metal ions. To investigate the performance of the composites in pH measurement, 4-MPy was applied as a Raman-active molecule.

## 2. Experimental Section

### 2.1. PS/Silicon Nanocrystal Containing Polymer (SiP) Synthesis and Characterization

Polystyrene microspheres (PS, diameter: 3 µm, Sigma-Aldrich, St. Louis, MO, USA) were applied as a solid template. Silicon nanocrystal containing polymer film was in situ synthesized on PS by the reaction of (3-aminopropyl)-trimethoxysilane (APTES, Sigma-Aldrich) and L-ascorbic acid (L-AA, Sigma-Aldrich). Briefly, 20 µL PS solutions were mixed with 2 µL APTES and 20 µL of 0.1 M L-AA aqueous solutions in 1 mL isopropanol alcohol (IPA, Sigma-Aldrich). The mixture was kept at 60 °C for 24 h. After reaction, the formed PS/SiP were first precipitated through centrifugation at 10,000× *g* for 5 min (Thermo Scientific, Waltham, MA, USA), then washed three times using IPA.

In order to obtain hollow SiP microspheres (SiPM), the prepared PS/SiP were suspended in toluene for 5 min to remove PS. After washing and centrifugation, hollow SiPM were obtained. They were synthesized with the same initial concentrations of APTES and L-AA, but without PS as templates. Scanning electron microscopy (SEM, JSM 7500F, JEOL, Tokyo, Japan), transmission electron microscopy (TEM, JEOL JEM 2100, Tokyo, Japan), and energy dispersion spectroscopy (EDS, JEM ARM 200F, JEOL, Tokyo, Japan) were applied for characterizing morphology and elements of products. In order to observe SiPS by TEM (JEOL JEM 2100, Tokyo, Japan), SiPS were cut into 70 nm thin slices by an ultramicrotome.

### 2.2. Hollow SiPM/Au Synthesis

For the preparation of SiPM/Au, 30 µL of 20 mM HAuCl_4_ (Sigma-Aldrich) was added in a hollow SiPM dispensed solution and reacted for 24 h. After reaction, the products were collected through centrifugation at 10,000× *g* for 5 min, washed by IPA three times, and finally stored in IPA. Free gold nanoparticles were prepared through the Fren’s method. In brief, 5 mL of 0.1% (*w*/*v*) HAuCl4 aqueous solution was refluxed to boiling under vigorous stirring. Then, 5 mL of 1% (*w*/*v*) sodium citrate aqueous solution was added quickly. The mixture was kept boiling for 30 min under stirring and cooled to room temperature.

### 2.3. SERS Sample Preparation and Spectra

In this work, 4-mercaptopyridine (4-MPy, Sigma-Aldrich) was used as a Raman reporter for SERS detections. Briefly, 4-MPy was dissolved in ethanol with various concentrations (ranging from 10^−2^ to 10^−7^ M) and stored in the dark before use. SiPM/Au composites were functionalized with 4-MPy to obtain SERS samples by adding 10 µL 4-MPy solution in a 990 µL SiPM/Au dispersion. For the 10^−3^ M sample, 10 µL 4-MPy solution was mixed with a 90 µL SiPM/Au dispersion. For the SERS measurements, 200 µL aliquots of the mixture were added to the sample cell.

All Raman spectra were obtained using a Raman spectrometer (Zolix, Beijing, China) with a 785 nm laser system operating at a power of 1 mW. The exposure time for each sample was 5 s accumulation.

## 3. Results and Discussion

### 3.1. PS/SiP Synthesis and Characterization

**Figure 1:**
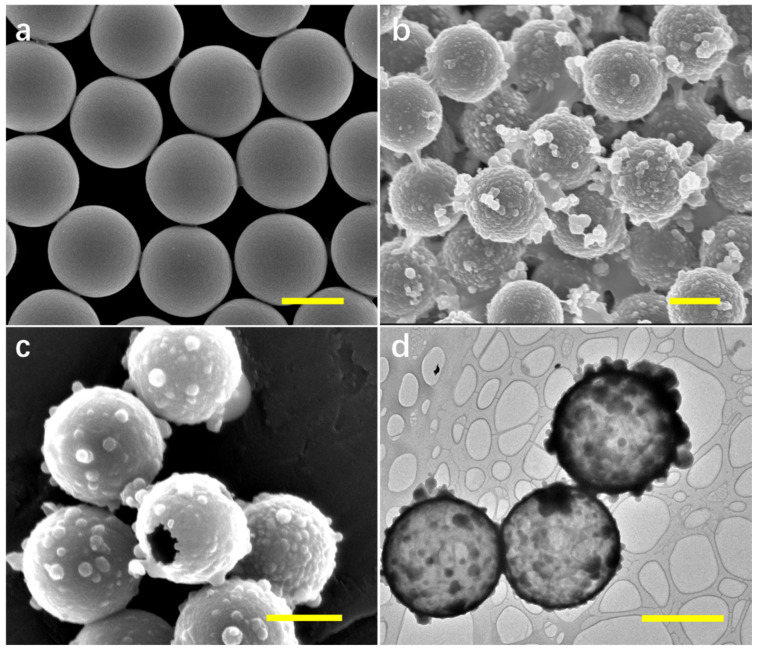
Scanning electron microscopy (SEM) image of (**a**) polystyrene microspheres (PS), (**b**) PS/silicon nanocrystal containing polymer (SiP) composites, and (**c**) hollow SiP microspheres (SiPM) ((3-aminopropyl)-trimethoxysilane (APTES)/ L-ascorbic acid (L-AA): 1/10). (**d**) Transmission electron microscopy (TEM) image of hollow SiPM. Scale bar: 2 µm.

Hollow silicon nanocrystal embedded microspheres (SiPM) were prepared using PS as solid templates. The morphology of PS, PS/SiP, and hollow SiPM was verified by SEM and TEM. The SEM image in Figure 1a shows PS with uniform morphologies (diameter: 3 µm). In order to prepare PS/SiP composites, PS were mixed with a precursor solution for SiPM containing APTES and L-AA. During this time, APTES was oxidized by L-AA in IPA and condensed SiP films were formed on PS, and the resulting products were collected by centrifugation and washed several times to remove unwanted smaller pure SiP particles. After reaction, the particle size increased by about 120 nm, indicating successful growth of SiP on PS (shown in Figure 1b). The prepared PS/SiP composites were then immersed in toluene for a few minutes to remove the PS template. Figure 1c shows the SEM image of hollow SiPM formed by removing PS inside. In order to confirm successful template removal, TEM was applied, and results are displayed in Figure 1d, verifying the formation of hollow SiPM structures. As shown in Figure S1 (supporting information), Si nanocrystals with small diameters (~2 nm) are well distributed in the polymer, which is observed by the high-resolution transmission electron microscop (HR-TEM) image. Green fluorescence is evenly distributed on the surface of hollow SiPM structures (shown in Figure S2). Both 2D and 3D confocal images confirm the hollow structure of SiPM. The SEM image of large scale hollow SiPM is shown in Figure S3, which confirms uniform morphologies of products.

**Figure 2:**
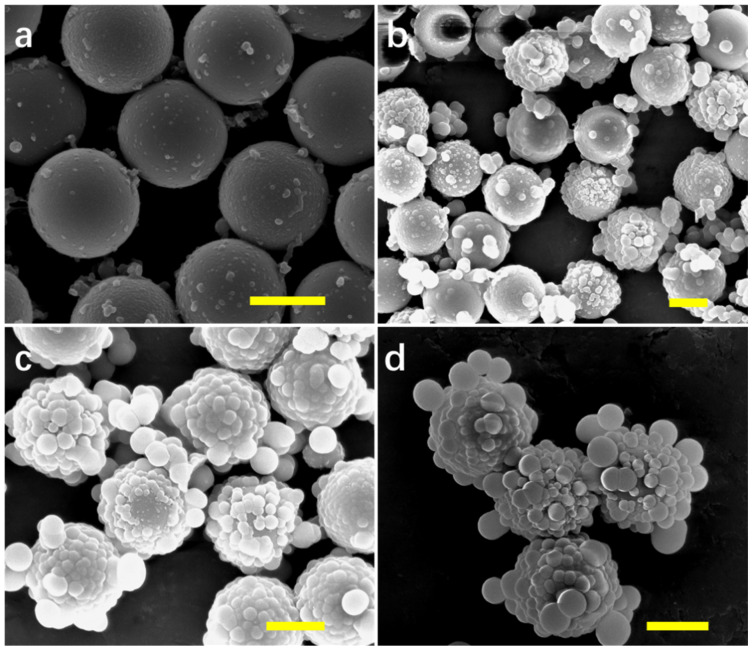
SEM images of PS/SiP composites synthesized with different initial reactant (APTES/L-AA) volume in the system. (**a**) 0.5/5, (**b**) 2/20, (**c**) 3/30, and (**d**) 4/40. Scale bar: 2 µm.

The effect of initial concentrations of APTES and L-AA in precursor solutions on the final products of SiP was investigated. The initial volume ratio of APTES to L-AA (0.1 M) was fixed while changing precursor concentrations in the system. After reaction, SiP was formed on the surface of PS with different film thickness. The morphologies of PS/SiP composites were observed by SEM. The initial reactant concentrations showed a great impact on final products. As shown in Figure 2, thicker SiP shells were formed on templates with increasing precursor concentrations. When the initial ratio of APTES/L-AA was 0.5/5 (shown in Figure 2a), the obtained composites showed a small morphology change compared with PS. The smooth PS surfaces were decorated with heterogeneous SiP particles. The PS templates were removed to verify whether a complete SiP film was formed. As shown in the TEM image (Figure S4), though hollow SiPM was obtained, the products could not maintain their spherical hollow structure. When the volume ratio of APTES/L-AA increased, uneven, thick SiP was generated on the PS. Lots of little balls were distributed on PS when the volume ratio of APTES/L-AA was more than 2/20 (Figure 2b–d). The average diameters of formed composites were close to 4 µm when the volume ratio of APTES/L-AA was 4/40, indicating SiP with thickness of 1 µm were formed on PS, which are too thick and not applicable for the next reduction of metal ions. Therefore, the ratio of 1/10 was applied for preparing hollow SiPM structures.

### 3.2. Hollow SiPM/Au Preparation

SERS has been a significant technology in biological detection. SERS signals are determined by “hot spots” and the distance between metal nanoparticles and analyte molecules. The structure of aggregated noble metal nanoparticles exhibits a high SERS effect compared with free nanoparticles. The hollow SiPM structure is suitable to develop surficial supported metal nanoparticles as SERS-effective substrates.

**Figure 3:**
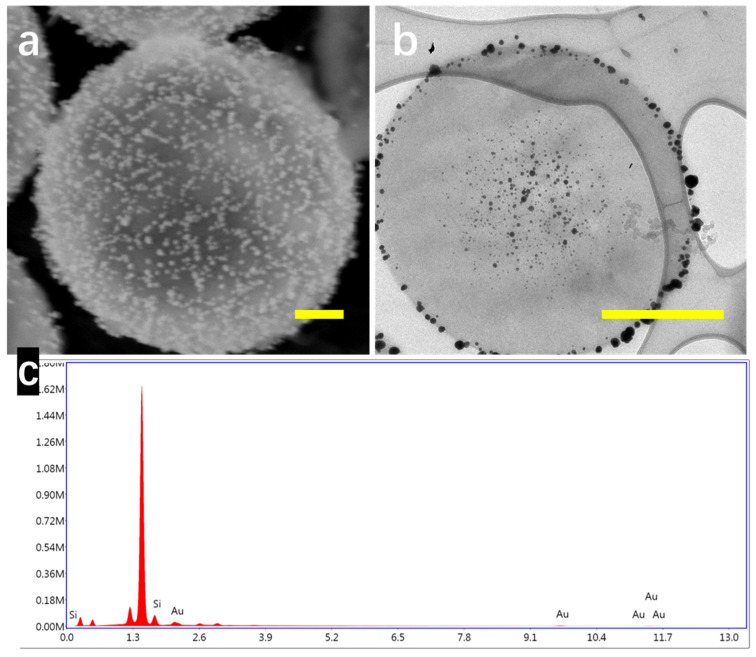
(**a**) SEM image of hollow SiPM/Au composite; (**b**) TEM image of SiPS/Au composite; and (**c**) EDS spectra of hollow SiPM/Au composite. Scale bar: 500 nm.

In order to achieve a SERS-active substrate, noble metal nanoparticles are formed on the surface of hollow SiPM structures. Previous research proved that SiPS are able to reduce noble metal ions into nanoparticles from their aqueous solutions because of the existence of L-AA in SiPS [18]. After mixing with HAuCl_4_ solutions, a lot of Au nanoparticles were formed and accumulated on the surface of hollow SiPM, as shown in Figure 3a. The size distribution histogram of gold nanoparticles and the extinction spectrum of SiPM/Au are shown in Figures S5 and S6, respectively. There was no excess of L-AA introduced in the process. Because of the porous structure of SiPM and the existence of APTES in SiPM proved by previous research, nanoscale gold nanoparticles were formed without the addition of stabilizer in the system. The coating ratio of Au nanoparticles was adjustable by changing the concentration ratio between SiPM and HAuCl_4_. However, for SiPS/Au composites, which have a smaller size and lack PS as templates applied during synthesis, Au nanoparticles are distributed not only on the outer surface but also in the nanopores at the center, due to uniform distribution of L-AA in SiPS, which is verified by the TEM image (shown in Figure 3b). The homogeneous distribution of Au on the surface and in the core of SiPS/Au composites could cause Au waste and SERS signal reduction. Element analysis of hollow SiPM/Au is shown in Figure 3c, verifying the presence of Si and Au in the composites.

### 3.3. SERS Spectra

**Figure 4:**
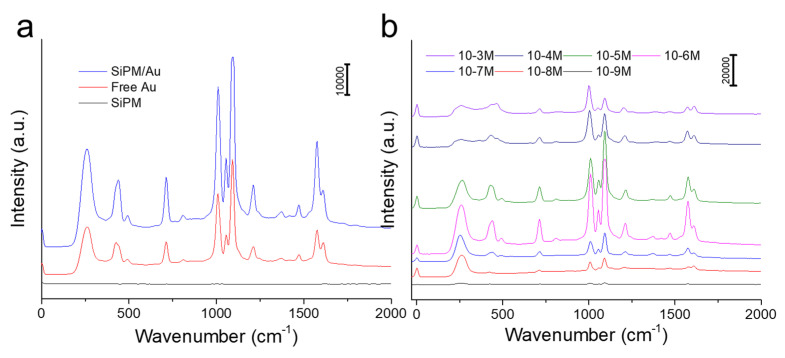
(**a**) SERS spectra of 10^−6^ M 4-MPy on SiPM, free Au, and SiPM/Au composites; (**b**) SERS spectra of 4-MPy on SiPM/Au with different concentrations (from 10^−3^ to 10^−9^ M).

The Raman spectra of typical SERS model molecules were frequently applied to evaluate the enhancement effect of the substrates. Here, 4-MPy was applied as a SERS signal molecule. A 785 nm laser was chosen to obtain SERS signal. The bare hollow SiPM and free Au nanoparticles with the same structures were used as controls. After mixing with 4-MPy (10^−6^ M), signals were detected and reflected with characteristic Raman shifts. Several characteristic Raman peaks, such as 1012, 1055, and 1607 cm^−1^, were obtained with a high signal from the spectra. As shown in Figure 4a, two strong SERS bands of 4-MPy at 1012 and 1093 cm^−1^ are displayed. No Raman signals were shown on bare hollow SiPM, even when higher concentrations of 4-MPy were used. Compared with free Au nanoparticles, hollow SiPM/Au showed higher intensity on spectra, which may be due to abundant “hot spots” on the SiPM/Au composites to attract more analytes.

Here, 4-MPy served as a pH-probe molecule. SERS spectra of 4-MPy with various concentrations from 10^−3^ to 10^−9^ M were measured to examine the sensitivity of hollow SiPM/Au structures. The 4-MPy molecules bound onto Au effectively via the Au–S bond. The detectable concentration of 4-MPy on SiPM/Au was as low as 10^−9^ M according to the results in Figure 4b. The Raman intensity dependence on concentrations of 4-MPy has been shown in Figure S7. According to previous reports [22], a low concentration of 4-MPy cannot be absorbed on the surface of SiPM/Au sufficiently, resulting in low Raman intensity, while an extremely high concentration might cause aggregation of SiPM/Au, also resulting in low Raman intensity. The 4-MPy with 10^−6^ M showed the highest Raman intensity, which might be due to optimum adsorption efficiency on hollow SiPM/Au composites. The enhancement factor (EF) is used to evaluate the SERS capability. The common formula for calculating the EF is shown in Formula (1).

(1) $$\mathrm{EF}=\frac{I_{SERS}/C_{SERS}}{I_{RS}/C_{RS}}$$

where $I_{SERS}$ is the Raman intensity obtained for the SERS substrate under a certain concentration. $C_{SERS}$ is the Raman label molecule. $I_{RS}$ is the Raman intensity obtained under non-SERS conditions at an analyte concentration of $C_{RS}$ (Figure S8). The peak intensity at 1095 cm^−1^ is applied as the reference. A value of EF is estimated to be 5.4 × 10^7^ for the SiPM/Au ($C_{SERS}$ = 1.0 × 10^−9^ M, $I_{SERS}$ = 1026, $C_{RS}$ = 1.0 × 10^−2^ M, and $I_{RS}$ = 189).

The reproducibility of SERS signals is essential for real application. The Raman spectra of 4-MPy for 20 different batches are displayed in Figure S9. The relative standard deviation (RSD) values of signal intensity at 1012 and 1093 cm^−1^ were calculated to be 6.08% and 5.04%, respectively, implying good reproducibility.

**Figure 5:**
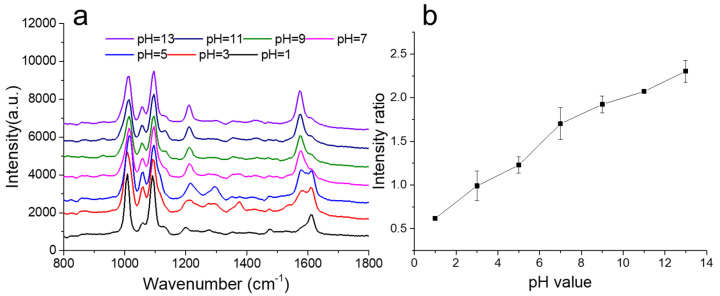
(**a**) SERS of 4-MPy (10^−6^ M) on SiPM/Au in phosphate buffered saline (PBS) with different pH values; (**b**) the intensity ratio variation of 1574/1607 cm^−1^ at different pH values.

The 4-MPy is the most frequently used molecule for pH sensing. The sulfhydryl group in 4-MPy can interact with Au sites on SiPM, forming a strong Au–S bond. When the 4-Mpy molecule is exposed to the sample with different pH values, the protonation and deprotonation of the molecule lead to obvious changes in the relative intensity of typical Raman peaks. The SERS spectra of 4-Mpy in phosphate buffered saline (PBS) solution with different pH values ranging from pH 3.0 to 9.0 are shown in Figure 5a. The peals at 1574 cm^−1^ were responsible for ring deformation with C=C antisymmetric stretch and 1607 cm^−1^ stands for ring deformation with C=C symmetric stretch, which are frequently chosen as indicators in SERS-based pH sensing. Here, the intensity ratio of 1574 cm^−1^ to 1607 cm^−1^ was utilized to monitor pH response. It is noted that the peak intensity at 1607 cm^−1^ decreased with increasing pH values. As pH values increased, the ring stretching mode of protonated forms decreased. The change of values of intensity ratio along with values of pH is displayed in Figure 5b. The intensity ratio of peak pairs such as 1574 vs. 1607 cm^−1^ varied with pH values. There was a linear response between intensity ratio and pH values, and a low standard deviation indicated good homogeneity of the samples.

The stability and reliability of hollow SiPM/Au structures are significant in pH sensing. The SERS spectra obtained from a prepared pH sensor after 7 days are displayed in Figure S10. It is demonstrated that no obvious change was observed in both peak position and intensity, which can be attributed to the stable structure of SiPM/Au. The composites overcame the limitation of Au nanoparticle aggregation with hollow SiPM as supporting substrates. Free Au gold nanoparticles were aggregated easily, which is not suitable for long-term application.

Various studies reported applications of gold nanoparticle coated hollow particles in SERS [23,24]. Traditional chemical modification of gold nanoparticles on substrates is complicated, and the products are not stable compared with the one-step synthesis method [11]. L-AA has been applied to reduce gold ions in the reaction system; however, many gold nanoparticles are not only formed on microspheres but also in solution [25]. Due to existence of L-ascorbic acid in SiPM, gold ions are directly reduced to gold nanoparticles, which simplify the synthesis process. In addition to the reducibility, the microcapsules show stable green fluorescence. The luminescence of silicon nanocrystals has expanded their applications because of their better biocompatibility and fluorescent stability. After interaction with metal ions, the formed hybrids show superior functions in both fluorescence and SERS, forming SERS–fluorescence dual-mode materials, which need further application.

## 4. Conclusions

In summary, a novel SERS substrate, SiPM supported Au nanoparticle composites, was prepared. Owing to the presence of hollow SiPM as supporting substrates, the SiPM/Au showed an enhanced SERS effect compared with free Au nanoparticles. The platform exhibited good SERS sensitivity with an EF value of 5.4 × 10^7^, and the detectable concentration of 4-MPy on SiPM/Au was as low as 10^−9^ M. The structure with SiPM substrate supported metal nanoparticles not only improved stability of gold nanoparticles but also exhibited good signal reproducibility, with an RSD of less than 6.1%. The composite showed a highly sensitive response to pH changes with 4-MPy as a signal molecule. The intensity ratio of 1574 to 1607 cm^−1^ showed linear response with pH values. The properties give the composites the potential of practical application in biological detection.

## Supplementary Materials

The following are available online at https://www.mdpi.com/2079-4991/10/8/1501/s1, Figure S1: HRTEM image of SiPM slices (70 nm, cut by ultramicrotome) with well-distributed silicon nanocrystals inside; Figure S2: (a) microscope and (b) confocal image of hollow SiPM structure; (c) 3D fluorescent confocal image of hollow SiPM structure; Figure S3: SEM image of large scale hollow SiPM; Figure S4: TEM image of hollow SiPM obtained with low concentration ratio of APTES/L-AA (0.5/5); Figure S5: the size distribution histogram of gold nanoparticles; Figure S6: The extinction spectra of SiPM/Au particles; Figure S7: The Raman intensity dependence on concentrations of 4-MPy on SiPM/Au; Figure S8: the Raman spectrum of 4-MPy at 0.01 M obtained under non-SERS conditions; Figure S9. (a). SERS spectra of 10-6 M 4-MPy collected from 20 random spots on SiPM/Au composites; (b). RSD of different predominate peaks conveying both intensity and reproducibility of 20 points; Figure S10: SERS spectra of 4-MPy on SiPM/Au composites at pH 7.0 before and after storage for 7 days.
