# Supplementary material for: Synthesis of Gold Nanoparticle Stabilized on Silicon Nanocrystal Containing Polymer Microspheres as Effective Surface-Enhanced Raman Scattering (SERS) Substrates
Source: Nanomaterials (Basel). 2020 Jul 31;10(8):1501. doi: 10.3390/nano10081501 (PMC7466634; doi:10.3390/nano10081501)
Supplement: Supplementary file 1 [file nanomaterials-10-01501-s001.pdf]

nanomaterials

MDPI

Article

Synthesis of Gold Nanoparticle Stabilized on Silicon Nanocrystal Containing Polymer Microspheres as Effective Surface-Enhanced Raman Scattering (SERS) Substrates

Guixian Zhu [1,*], Lin Cheng [1], Gannan Liu [1] and Lianqing Zhu [1,2,*]

[1] School of Instrument Science and Opto-electronics Engineering, Beijing information Science and Technology University, Beijing 100192, China; chenglin51720@163.com (L.C.); 17810257075@163.com (G.L.)
[2] School of Instrument and Opto-electronics Engineering, Hefei University of Technology, Anhui 230009, China
* Correspondence: zhuguixian@bistu.edu.cn (G.Z.); zhulianqing@bistu.edu.cn (L.Z.)

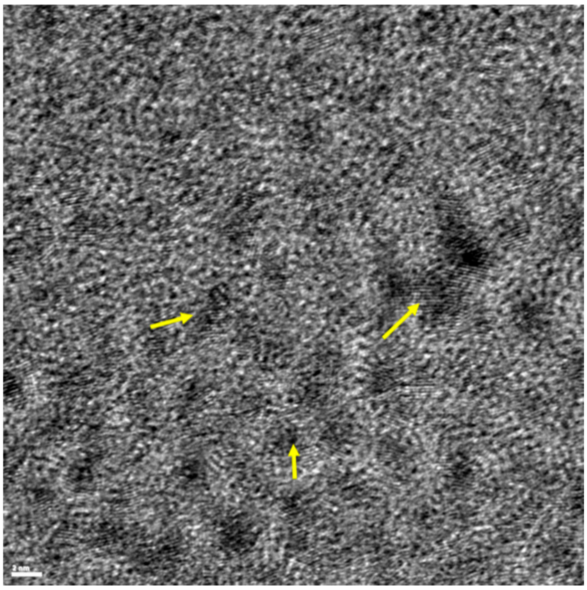

Figure S1. HRTEM image of SiPM slices (70 nm, cut by ultramicrotome) with well-distributed silicon nanocrystals inside.

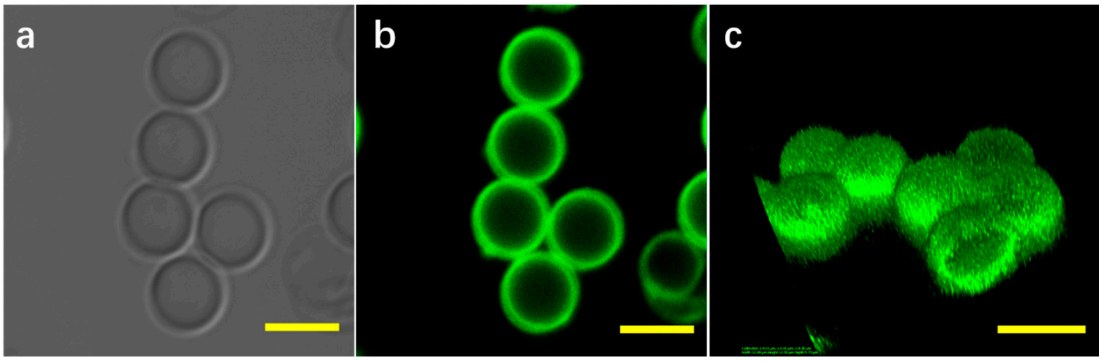

Figure S2. (**a**) microscope and (**b**) confocal image of hollow SiPM structure; (**c**) 3D fluorescent confocal image of hollow SiPM structure. Scale bar is 3 μm for all images.

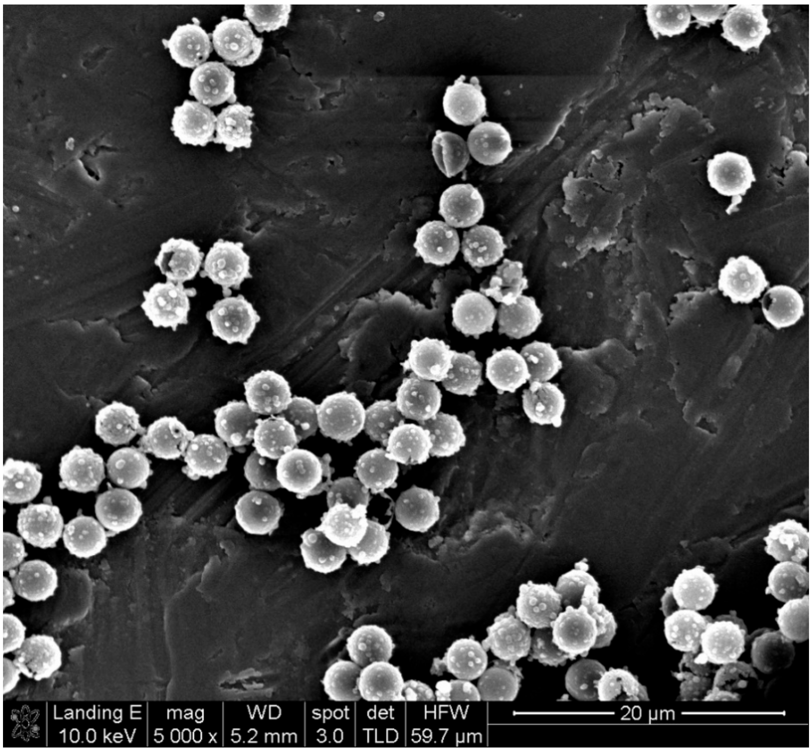

Figure S3. SEM image of large scale hollow SiPM.

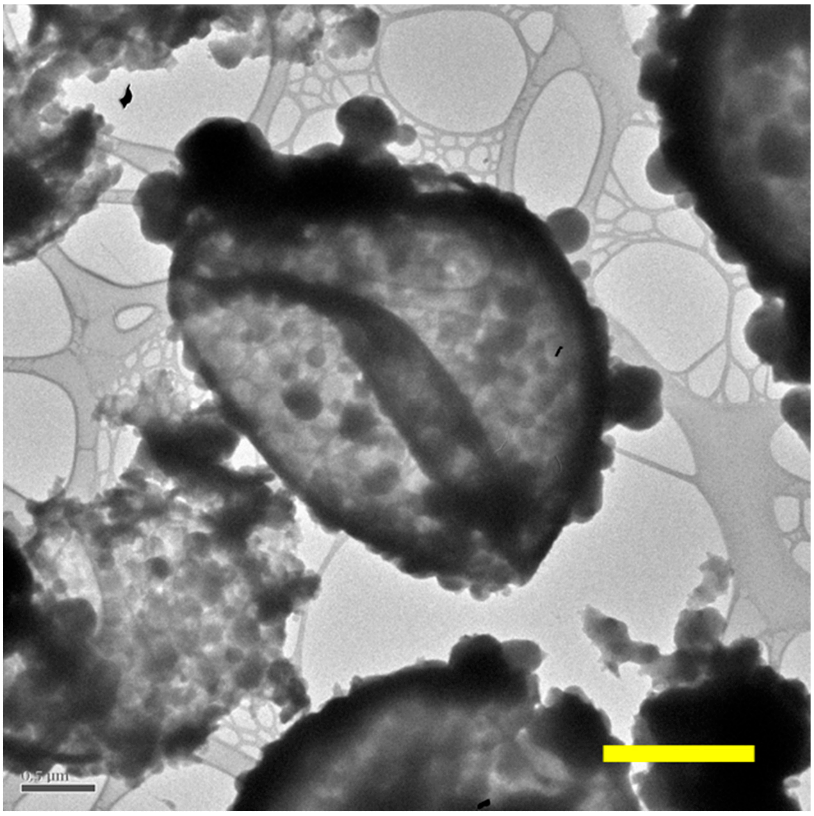

Figure S4. TEM image of hollow SiPM obtained with low concentration ratio of APTES/L-AA (0.5/5). Scale bar: 1 μm.

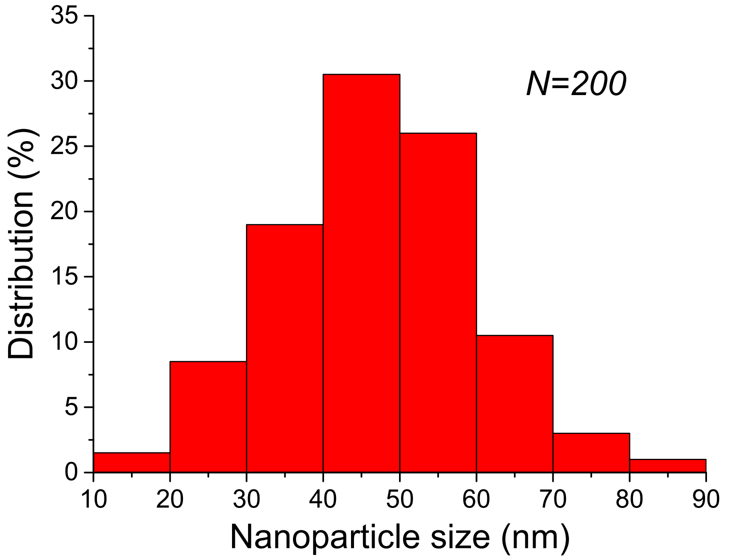

Figure S5. the size distribution histogram of gold nanoparticles.

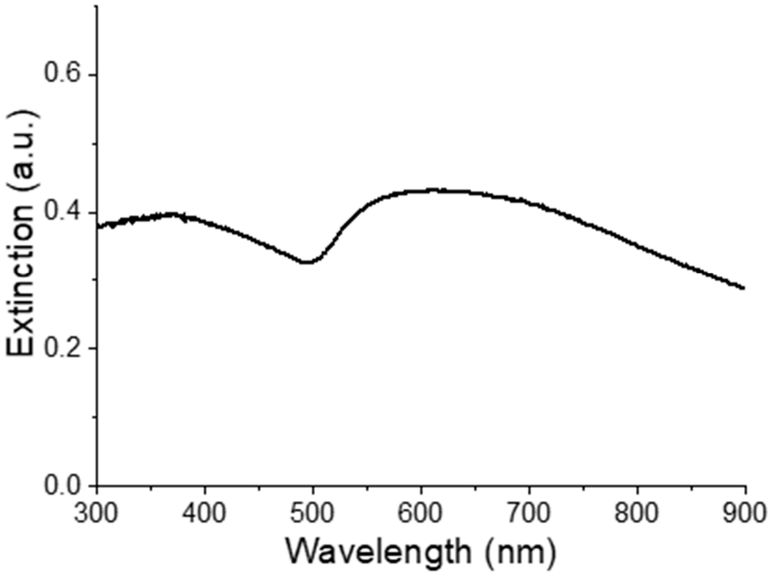

Figure S6. The extinction spectra of SiPM/Au particles.

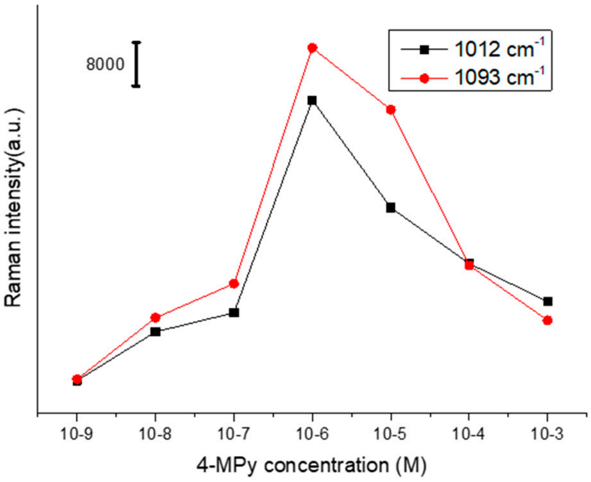

Figure S7. The Raman intensity dependence on concentrations of 4-MPy on SiPM/Au.

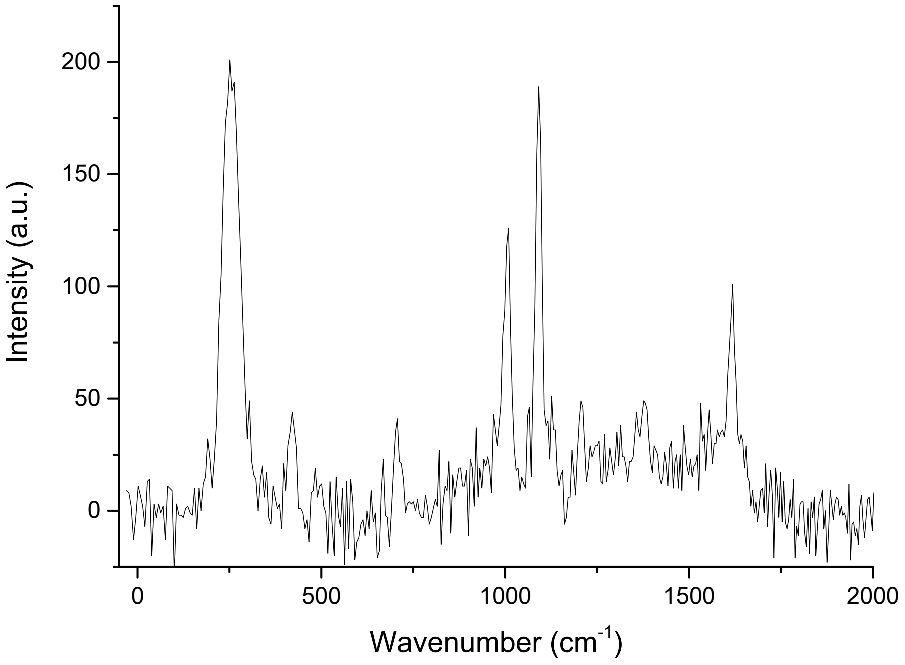

Figure S8. The Raman spectrum of 4-MPy at 0.01 M obtained under non-SERS conditions.

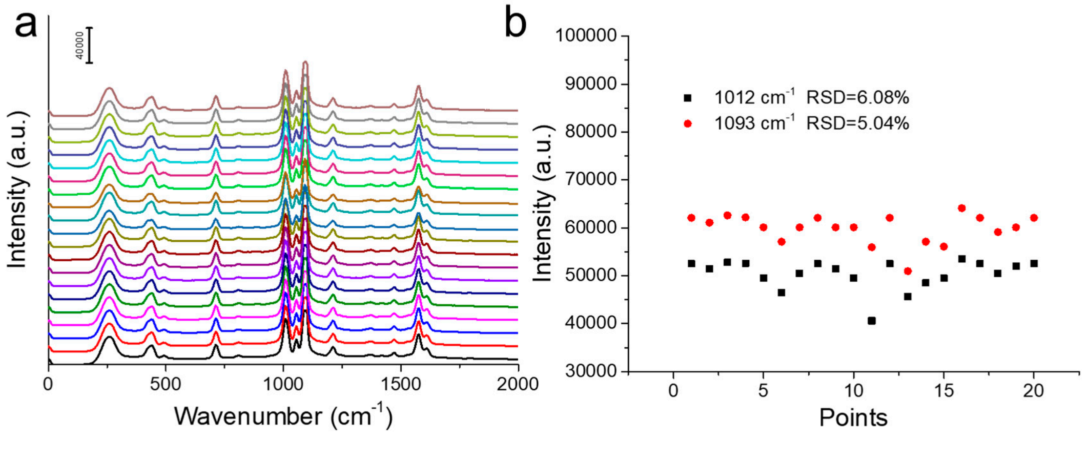

Figure S9. a. SERS spectra of 10^{-6} M 4-MPy collected from 20 random spots on SiPM/Au composites; b. RSD of different predominate peaks conveying both intensity and reproducibility of 20 points.

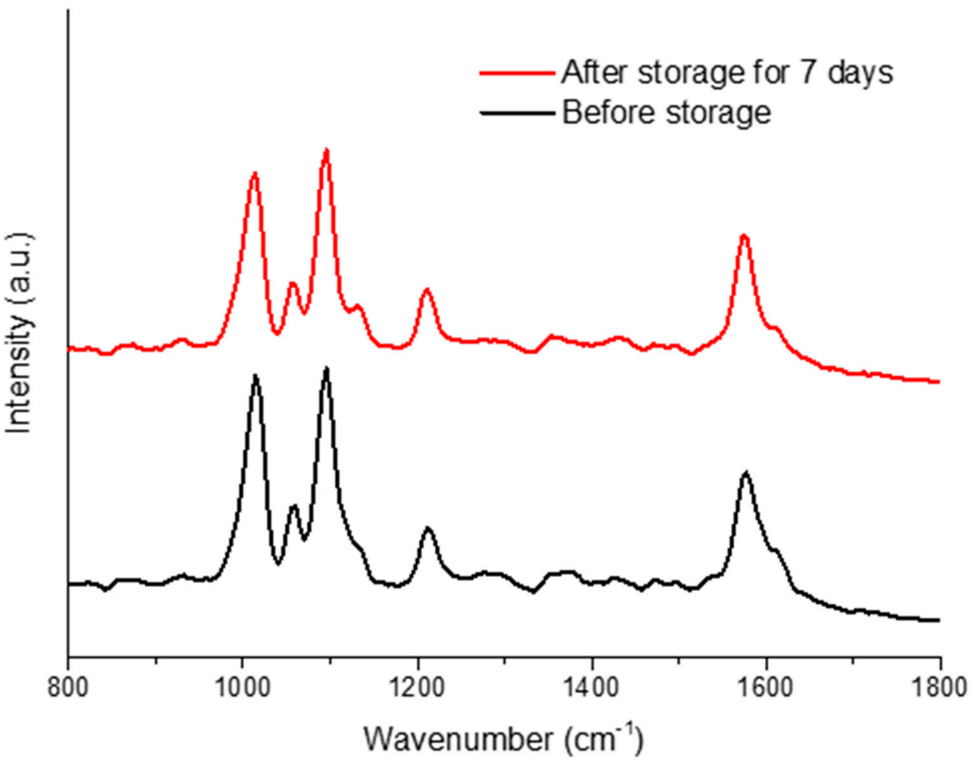

Figure S10. SERS spectra of 4-MPy on SiPM/Au composites at pH 7.0 before and after storage for 7 days.
